# A Surgeon's Perspective of Abdominal Wall Endometriosis at a Caesarean Section Incision: Nine Cases in a Single Institution

**DOI:** 10.1155/2014/765372

**Published:** 2014-09-22

**Authors:** Eun Mee Oh, Won-Suk Lee, Jin Mo Kang, Sang Tae Choi, Keon Kuk Kim, Woon Kee Lee

**Affiliations:** Department of Surgery, Gachon University Gil Hospital, 1198, Guwol-dong, Namdong-gu, Incheon 405-760, Republic of Korea

## Abstract

Abdominal wall endometriosis in a Caesarean section scar (AEC) is an infrequent type of extrapelvic endometriosis which rarely transforms into a malignant lesion. A painful mass located in the scar of a Caesarean section is a typical sign of AEC. This condition is diagnosed preoperatively using imaging modalities such as computed tomography and ultrasonography, as well as fine-needle aspiration. Although AEC has typical signs, general surgeons often misdiagnose it due to its rarity. Herein, we report our experience of AEC in a single institution.

## 1. Introduction

Extrapelvic endometriosis, an uncommon form of the disease, can affect unusual sites including the urinary tract, gastrointestinal tract, and thorax. The incidence of abdominal wall endometriosis in a previous Caesarean section (C/S) scar has been reported at ~1-2% in patients undergoing lower abdominal surgery [1]. Despite its rarity, several reports on abdominal wall endometriosis in a Caesarean section scar (AEC) have been published. A tender and painful abdominal wall mass is considered suggestive of AEC in females of reproductive age with a previous history of C/S [1, 2]. Imaging studies including ultrasonography (USG) and computed tomography (CT) can aid the diagnosis of AEC; however, the condition is often misdiagnosed and referred to general surgeons [3]. Herein, we report nine cases of AEC from the perspective of a general surgeon.

## 2. Case Report

The study data were gathered from the electronic medical records from the period 2002 to 2013, during which a total of nine cases of AEC were confirmed histologically. Among them, six female patients presented to general surgeons and the remaining three visited a gynaecology clinic due to their chief complaint. Nine clinicians, including three gynaecologists and six general surgeons, evaluated and treated these nine patients.

All patients were premenopausal and between 29 and 40 years of age. Five patients had undergone C/S twice and the remaining four patients had had one C/S. One of the patients had a history of preeclampsia and the other eight had no notable obstetric or gynaecologic history.

Pfannenstiel skin incisions and tender masses were found in the previous C/S scars of all patients. In four of the patients, menstruation was associated with tenderness in the C/S scar. The duration of clinical symptoms varied from 3 months to 3 years.

Computed tomography (CT) was used in five patients and ultrasonography (USG) was used in three patients to evaluate the lesions. No imaging was used in the remaining patient, but the first impression of the lesion by physical examination was abdominal wall endometriosis.

Only one general surgeon and two gynaecologists suspected AEC initially prior to imaging modalities such as CT and USG. One gynaecologist diagnosed an unknown subcutaneous mass, while the remaining five general surgeons suspected other disease entities including a desmoid tumour, epidermal cyst, or postoperative granuloma.

An excisional biopsy was performed under general anaesthesia in six patients, under spinal anesthesia in two patients, and under local anaesthesia only in one patient. The sizes of the lesions varied from 1 to 7 cm and a closed suction drain was inserted into the surgical site in two patients.  Table 1 shows the clinical data of all patients.

**Figure 1:**
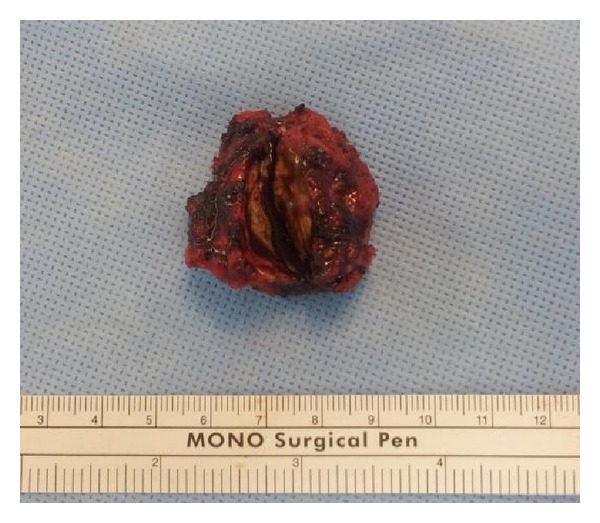
A mass of ~3.5 cm was removed. Grossly, white-yellowish-coloured tissue and brownish tissue were observed inside the lesion. A dark-brown-coloured fluid was found upon incision of the lesion.

**Figure 2:**
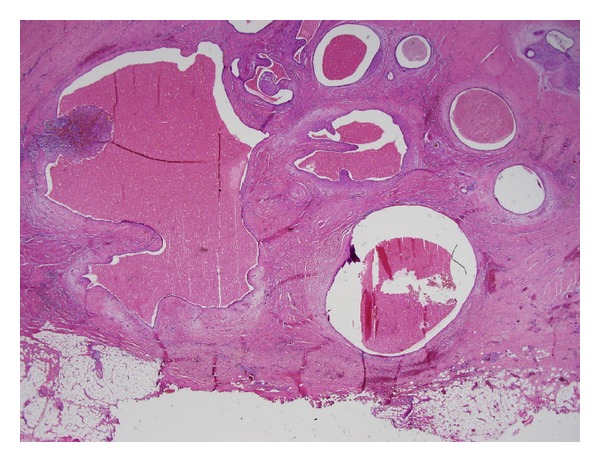
Endometrial glands, stroma, and adipose tissue were found within the connective tissue. H&E staining, ×10 magnification.

Each patient underwent one followup on an outpatient basis after 2 weeks postoperatively; no postoperative complications, including surgical site infection or haemorrhage, were observed. No further followup was planned in any patient. In all patients, endometriosis was confirmed histologically. Grossly, light-grey to light-brown soft tissue was observed on cross sections and endometrial glands and stroma were identified microscopically (Figures 1 and 2).

## 3. Discussion

Endometriosis is a benign disease characterised by normal endometrial tissue outside the uterine cavity. Extrapelvic endometriosis can be found intra-abdominally as well as in the abdominal wall. In one previous report, endometriosis in the abdominal wall was related to a previous history of surgery, and Emre et al. reported one case of abdominal wall endometriosis in a laparoscopic trocar port site [4]. The disease has also been recorded without history of a previous surgery [5].

Abdominal wall endometriosis occurring in a C/S scar is very rare, so few reports are available in the literature. According to one report, the incidence of AEC ranges from 0.2% to 0.45%. However, almost all studies on AEC are limited by the small number of cases due to the rarity of this disease [6].

Studies on risk factors for AEC are also scarce, but Caesarean section is considered a risk factor for the condition [6]. Furthermore, other risk factors include an early hysterotomy in pregnancy, increased menstrual flow, and alcohol consumption. High parity is known to be protective against AEC [7].

A painful mass that may or may not be related to the menstrual cycle is pathognomonic for AEC. In the present report, 80% of patients complained of pain, which waxed and waned with the menstrual cycle in 40%. The average time between C/S and the onset of clinical symptoms was reported to be 3.7 years [7].

The usefulness of imaging studies, including CT and USG, as well as fine-needle aspiration cytology (FNAC) is well documented. On ultrasound, AEC appears as a solid, heterogeneous hypoechoic mass with inner echogenic spots. The echogenic patterns are dependent on the haemorrhagic and fibrous components of the lesions. Although the attenuation varied, on CT mild-to-moderate enhancement of the lesion was observed in the abdominal wall close to the C/S scar [3]. Medeiros et al. published their clinical experience of FNAC in nine cases of AEC. They identified clusters of epithelial endometrial-like cells, endometrial-like stromal cells, and haemosiderin-laden macrophages in the lesion. Therefore, they concluded that FNAC is an inexpensive, rapid, and accurate diagnostic tool for detecting AEC [8].

Medical therapy can be used to relieve the clinical symptoms of AEC and often involves hormone suppression to downregulate the hypothalamus-pituitary-ovarian axis, but if ineffective, surgical excision may be required. Surgery is the definitive treatment option for preventing recurrence of AEC and conversion to malignancy, although this event is very rare [1, 2, 9]. Abdominoplasty and reconstruction with or without polypropylene mesh should be considered if a defect in the abdominal wall occurs, which may be caused by wide excision of the AEC [10].

In this case series, clinical data were retrospectively collected from the electronic medical records in a single institution. Therefore, the sample size was too small for identification of the incidence and risk factors of AEC. However, pathognomonic clinical symptoms of AEC were present in almost all of the patients, with two exceptions. An appropriate evaluation using imaging studies including CT and USG was performed in eight patients, but FNAC was not used as a diagnostic tool for AEC.

Interestingly, five general surgeons did not suspect AEC preoperatively. Of these five cases, one had a very small (1.7 cm) lesion but no other clinical symptoms except mild tenderness. In this case, there was an impression of reactive lymph node hyperplasia on USG but AEC was difficult to diagnose preoperatively. However, the remaining four patients complained of a painful mass related to the menstrual cycle as the pathognomonic clinical symptom. The four general surgeons who examined these patients did not suspect AEC. Their first impressions at the outpatient department were other nongynaecologic diseases since they do not typically collect obstetric and gynaecologic information during the medical examination. General surgeons see proliferative lesions that develop at an incision site (e.g., desmoid tumours, granulomas) far more often than AEC. One gynaecologist did initially suspect AEC after physical examination of a patient who had mild pain unrelated to her menstruation.

Although AEC is a rare entity not often seen in a general surgery clinic, general surgeons should be mindful of the possibility of AEC in patients with a painful mass in the C/S incision site that may be associated with the menstrual cycle.

## Figures and Tables

**Table 1:** Basic clinical information of all nine cases.

Patient number	Age at initial diagnosis	Year of C/S	Chief complaint	Department of the operator	First impression after physical examination	Imaging study used for diagnosis	Year of operation for AEC	Method of anesthesia	Obstetric history
1	31	2005	Painful mass has relationship with menstruation	GS	R/O desmoid tumor	CT	2008	G/A	1-0-0-1
2	34	2003	Painful mass has relationship with menstruation	GS	R/O desmoid tumor	CT	2007	G/A	2-0-0-2
3	40	2003	Painful mass	GS	R/O epidermal cyst	USG	2011	L/A	1-0-1-1
4	32	2002	Painful mass	GYN	R/O endometriosis	CT	2011	G/A	3-2-1-2
5	35	2002	Painful mass has relationship with menstruation	GYN	R/O endometriosis	USG	2010	G/A	1-0-0-1
6	29	2008	Painful mass has relationship with menstruation	GYN	R/O endometriosis	USG	2013	G/A	1-1-0-1
7	31	Unknown	Painful mass has relationship with menstruation	GS	R/O endometriosis	Not done	2005	S/A	2-0-0-2
8	36	2001	Painful mass has relationship with menstruation	GS	R/O desmoid tumor	CT	2007	S/A	2-0-1-2
9	32	2004	Painful mass has relationship with menstruation	GS	R/O granuloma in postoperative scar	CT	2013	G/A	1-0-1-1

C/S: Cesarean section; AEC: abdominal wall endometriosis in C/S scar; GS: general surgery; GYN: gynecology; G/A: general anesthesia; CT: computed tomography; USG: ultrasonography; L/A: local anesthesia; S/A: spinal anesthesia.

## References

[1] Mistrangelo M, Gilbo N, Cassoni P (2014). Surgical scar endometriosis. *Surgery Today*.

[2] Horton JD, DeZee KJ, Ahnfeldt EP, Wagner M (2008). Abdominal wall endometriosis: a surgeon's perspective and review of 445 cases. *The American Journal of Surgery*.

[3] Gidwaney R, Badler RL, Yam BL (2012). Endometriosis of abdominal and pelvic wall scars: multimodality imaging findings, pathologic correlation, and radiologic mimics. *Radiographics*.

[4] Emre A, Akbulut S, Yilmaz M, Bozdag Z (2012). Laparoscopic trocar port site endometriosis: a case report and brief literature review. *International Surgery*.

[5] Tomás E, Martín A, Garfia C (1999). Abdominal wall endometriosis in absence of previous surgery. *Journal of Ultrasound in Medicine*.

[6] Nominato NS, Prates LFVS, Lauar I, Morais J, Maia L, Geber S (2010). Caesarean section greatly increases risk of scar endometriosis. *European Journal of Obstetrics Gynecology and Reproductive Biology*.

[7] de Oliveira MAP, de Leon ACP, Coutinho Freire E, de Oliveira HC (2007). Risk factors for abdominal scar endometriosis after obstetric hysterotomies: a case-control study. *Acta Obstetricia et Gynecologica Scandinavica*.

[8] Medeiros FDC, Cavalcante DIM, da Silva Medeiros MA, Eleutério J (2011). Fine-needle aspiration cytology of scar endometriosis: study of seven cases and literature review. *Diagnostic Cytopathology*.

[9] Shalin SC, Haws AL, Carter DG, Zarrin-Khameh N (2012). Clear cell adenocarcinoma arising from endometriosis in abdominal wall cesarean section scar: a case report and review of the literature. *Journal of Cutaneous Pathology*.

[10] Collins AM, Power KT, Gaughan B, Hill AD, Kneafsey B (2009). Abdominal wall reconstruction for a large caesarean scar endometrioma. *Surgeon*.

